# The effect of changes in lower limb pain on the rate of progression of locomotor disability in middle and old age: Evidence from the NorStOP cohort with 6-year follow-up

**DOI:** 10.1016/j.pain.2011.12.006

**Published:** 2012-05

**Authors:** Sara Muller, Elaine Thomas, George Peat

**Affiliations:** Arthritis Research UK Primary Care Centre, Keele University, UK

**Keywords:** Locomotion, Disability evaluation, Pain, Longitudinal studies

## Abstract

Locomotor disability (LMD) is common at older ages, and can lead to other significant disability and mortality. Prevalent pain has been shown to be associated with LMD. This article aimed to assess the association between changes in lower limb pain status (ascertained from a manikin) and changes in the level of self-reported LMD in a sample of UK adults age ⩾50 years, over a 6-year period (data collected at 3-year intervals). There was an average increase in the level of LMD over 6 years. Reports of an onset of lower limb pain were associated with a relative increase in LMD, independently of sociodemographic factors and the onset of selected comorbid diseases. A dose-response relationship was observed between the onset of multiple lower limb joint involvement and more frequent or intense pain and larger increases in LMD. Becoming free from lower limb pain was associated with a relative decrease in LMD, but did not return LMD scores to the level of those who had remained pain-free throughout. This is consistent with a cumulative effect on LMD of recurrent episodes of pain. Lower limb pain may be a key target for prevention and rehabilitation to reduce years lived with disability in later life.

## Summary

Lower limb pain is an important determinant of locomotor disability. Recurrent episodes of pain may have a cumulative effect on functional decline in later life.

## 1 Introduction

Locomotor disability (LMD), defined here as, “walking, stair climbing and general getting about” [30], is the most common form of disability in community-dwelling older adults [16,26] and is associated with impaired quality of life [3,8]. It is often the first type of disability to occur [13], a precursor to other disabilities, morbidities, and mortality [13,17,20,28,42], with markedly higher prevalence at older ages [29].

Worldwide, life expectancies continue to increase, with no indication that the maximum possible life span has been reached [9]. Although there is some evidence that successive birth cohorts may be less likely to experience severe disability at a given age, the absolute numbers in the population with multidimensional health problems, many associated with LMD, are likely to increase.

In addition to chronological age, a wide range of determinants of LMD occurrence has been reported, including lower socioeconomic status [1,2,10,11,15,17,19,20,23,24,28,42], lifestyle factors [2,5,6,10,11,15,23,24,28,42], and long-term health conditions [5,11,15,19,42]. The group of health conditions considered most frequently in relation to LMD are those relating to musculoskeletal pain, in particular arthritis and lower limb pain, whose presence has been shown to precede and increase the subsequent rate of onset of LMD [15,19,33,42]. LMD can be measured from either a self-reported perspective or a performance-based perspective. Despite potential conceptual differences between these 2 ways of measuring LMD, the same association with pain has been shown to exist [10,11,15,25,33,42] and to persist after adjustment for environmental factors and comorbidities [10,27]. Indeed, Leveille et al. [25] suggest that the association between pain and LMD may be a direct one.

Knowledge of the precise nature of this relationship, however, remains incomplete. First, LMD has typically been studied as a dichotomous phenomenon (disabled versus not disabled). Yet in reality, LMD is likely to be a continuum. Studying changes in LMD across a spectrum of disability may provide additional insights compared to those based on the proportion of individuals crossing an arbitrary threshold [39]. Second, although pain is understood to vary over time [14,21], most previous studies have considered only prevalent pain and its association with the subsequent onset of LMD [10,11,15,25,42]. To fully understand the association between changes in lower limb pain status and changes in LMD, it is necessary to consider the effect of the onset of and recovery from pain on the level of LMD an individual experiences over time.

In this study, using a measure of LMD that captures its natural continuum [30], we sought to extend current evidence on the relationship between lower limb pain and LMD in adults age ⩾50 years. We hypothesised that (1) the onset of lower limb pain will result in a concurrent increase in LMD level over a 3-year period; (2) recovery from lower limb pain will result in a relative decrease in LMD; (3) there is a dose-response association between the characteristics of lower limb pain (frequency, intensity, number of joint sites affected) and changes in LMD, and this association is independent of potentially confounding factors (sociodemographic characteristics and the onset of selected comorbid diseases).

## 2 Methods

### 2.1 Populations

Data are taken from the North Staffordshire Osteoarthritis Project (NorStOP) [35], a prospective cohort study of community-dwelling adults. The sampling frame for the study was the registered population of 3 general practices in North Staffordshire, UK, age ⩾50 years in April 2002 (n = 11,309). A total of 7878 participants were recruited via a postal survey at baseline [36]. This questionnaire collected information on general health and sociodemographic information, as well as pain and disability. Baseline participants who gave consent to be contacted again, were known to still be alive, and with correct contact details were mailed further questionnaires 3 and 6 years later (April 2005 and April 2008). Respondents at 3 years (n = 4234) have previously been compared with respondents at baseline [37]. Here, 6-year respondents were compared with the original baseline cohort in terms of age, gender, socioeconomic status, and general health.

### 2.2 Ethical approvals

All participants provided written informed consent to take part in the study. Ethical approval was obtained from the North Staffordshire Local Research Ethics Committee (approval codes 1351 and 05/Q2604/20).

### 2.3 Sociodemographic characteristics

Gender and age at baseline were ascertained from the general practice records and verified by self-report. Participants reported their socioeconomic status at baseline in 3 ways. Educational attainment was derived from the question, “Did you go on from school to full time education or university?”. Possible responses were yes (“further education”) and no (“school-age education only”). Occupational class was assessed at baseline using current job title (most recent job for those who were not working) and classified according to the Standard Occupational Classification [31,32]. These were then regrouped into manual (lower supervisory/technical, semiroutine occupations, routine occupations) and nonmanual (higher managerial, higher professional, lower managerial/professional, intermediate occupations) occupations. Perceived adequacy of income was assessed using the item “Thinking about the cost of living as it affects you, which of these descriptions best describes your situation?: Find it a strain to get by from week to week; Have to be careful with money; Able to manage without much difficulty; Quite comfortably off” [38]. These responses were dichotomised into inadequate (“find it a strain to get by from week to week”, “have to be careful with money”) and adequate (“able to manage without much difficulty”, “quite comfortably off”).

### 2.4 Locomotor disability

LMD was measured using an interval-level score obtained from the walking and stair-climbing items of the Short Form-36 Physical Functioning subscale [43]. This score has been extensively tested in several datasets and shown to be repeatable and valid in the measurement of LMD [30]. Scores on this measure were obtained in this sample for all time points (baseline, 3- and 6-year follow-ups) simultaneously, using the RUMM2020 Rasch analysis program [4]. Scores were transformed to range from 0 (least LMD on scale) to 8.795 (most LMD on scale).

### 2.5 Lower limb pain trajectories

Lower limb pain was assessed using a screening item and a body manikin. The screening item asked, “In the past 4 weeks, have you had pain that has lasted for one day or longer in any part of your body?” with possible responses of yes and no. Front and back views of the body manikin were displayed, and respondents were asked to shade where they had pain. Standard transparent templates with the borders marked were used to assess in which area(s) of the body respondents had reported pain. This method has been shown to be repeatable (kappa values across 8 raters for regions of the lower limb: 0.86 to 1.00) [22]. Those responding no to the screening item and not shading any area of the manikin were considered not to have pain. Those shading the manikin were considered to have pain in the shaded area, regardless of their response to the screening item. Pain sites were divided into those in the lower limb (including the hips [7]) and the upper body (Appendix, Figure A). These data were collected at each of the 3 time points, and 8 trajectories of lower limb pain were defined (Table 1).

### 2.6 Lower limb pain characteristics

The lower limb pain characteristics at 3-year follow-up were considered at 3 sites: hip, knee, and foot. At each site, data were collected on the intensity of pain (0 to 10 numerical rating scale) and the frequency of pain (no pain days, 1 to 30 pain days, 31 to 89 pain days, 90 or more pain days) in the previous 6 months [41]. The overall lower limb pain intensity was defined as the highest intensity reported across the 3 sites. Similarly, the overall frequency of pain was defined as the highest frequency reported across all 3 sites. The number of pain sites in the past 6 months (0 to 3) was calculated as the number of sites with reported pain intensity >0.

### 2.7 Comorbid disease

At 3- and 6-year follow-ups, respondents were asked to report whether they had comorbidities from a prespecified list that included chest problems, eyesight problems (excluding the need for glasses), and leg pain on walking. Participants also completed the Hospital Anxiety and Depression Scale (HADS) [45]. A HADS score for depression of 11 or more was considered to indicate that a participant was depressed.

### 2.8 Statistical analysis

Tobit regression analysis was used to estimate mean (95% confidence interval) level of LMD at baseline and at 3- and 6-year follow-ups, overall and across the 8 lower limb pain trajectory groups. This technique allows for the skewed nature of the LMD score at each time point [40]. Regression models were adjusted for age group (50 to 59 years, 60 to 69 years, 70 to 79 years, ⩾80 years), gender, and baseline socioeconomic status (educational attainment, occupational class, and perceived adequacy of income). LMD scores at 3- and 6-year follow-ups were adjusted for score at baseline and 3-year follow-up, respectively, using appropriate fractional polynomial functions [18], to allow for any nonlinear association between LMD at the 2 follow-up time points.

As more detailed information regarding the presence of comorbidities and the characteristics of pain at specific lower limb sites was available at the 3- and 6-year follow-ups, further investigations into the potentially causal association between the onset of lower limb pain and changes in LMD were carried out in this time period. For this analysis, the onset (or not) of lower limb pain in the 2 time periods (baseline to 3-year follow-up; 3- to 6-year follow-up) are considered to be analogous due to the arbitrary nature of the timing of the data collection in relation to participants’ pain. Hence this analysis is carried out in the 3- to 6-year follow-up period, i.e., participants were free of lower limb pain at 3 years and either had or did not have an onset of lower limb pain at 6 years.

First, adjustment was made for the concurrent onset of morbidities (chest problems, visual impairment, leg pain on walking, depression, and pain in the upper body) previously shown to be cross-sectionally associated with LMD and lower limb pain [27] and hence considered to be possible confounders in the association between the onset of lower limb pain and changes in LMD. As these morbidities are generally considered to be chronic and as such recovery from them is unlikely, adjustment was made only for the onset of the morbidity that occurred between the 3- and 6-year follow-ups.

Second, to establish whether there was an association between the characteristics of the new-onset lower limb pain with changes in LMD, the presence of pain at the 3 lower limb joint sites (hip, knee, foot) at 6-year follow-up was considered. Initially, the presence of pain in any of the 3 lower limb joint sites or not (based on the manikin) was modelled (i.e., no lower limb pain sites at 6 years versus 1 to 3 sites) in those without lower limb pain at 3 years. Then, in those free of lower limb pain at 3-year follow-up, the 3 lower limb pain characteristics at 6-year follow-up (number of pain sites, pain intensity, pain frequency) were each modelled separately as potential predictors of change in LMD between 3- and 6-year follow-ups. The number of pain sites and intensity of pain were modelled using appropriate fractional polynomial functions. Three models were derived for each of any pain at 6 years and the 3 pain characteristics: a) adjusted for LMD at 3-year follow-up; b) additional adjustment for age group, gender, baseline socioeconomic status; and c) additional adjustment for the onset of comorbidities from 3- to 6-year follow-up. All analyses were carried out in Stata 11.

## 3 Results

### 3.1 Response

A total of 5129 potentially eligible participants responded to the baseline survey in March 2002, completed measures of lower limb pain and LMD, and consented to further contact (45.4% of total mailed population) [36]. Of these, 4234 responded at 3 years and 2831 at 6 years, with 2506 providing complete LMD and lower limb pain data at all 3 time points. Those people remaining in the cohort were more likely to be female, younger, belong to higher socioeconomic groups, and have slightly better physical and mental health scores than those who were not followed up (nonresponders, no consent to follow-up, deaths, and exclusions) (Table 2).

### 3.2 Lower limb pain trajectories

The most common trajectory was that of lower limb pain at all 3 time points (Table 1). This group had the highest proportion of female subjects and those in lower socioeconomic groups. It was also, on average, the oldest group.

### 3.3 Association of changes in locomotor disability with lower limb pain status

**Fig. 1:**
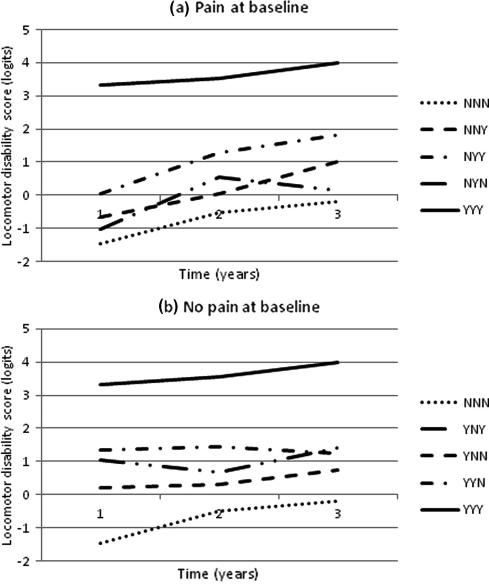
Changes in locomotor disability over 6 years according to lower limb pain status at baseline and at 3- and 6-year follow-up: results from Tobit regression model (means may be estimated outside range of original locomotor disability score).

Mean LMD scores over the 6-year study period, derived from the Tobit regression, are presented for each of the 8 trajectories of lower limb pain. For reference, the 2 extreme trajectories (NNN and YYY) are shown in both figures and those without baseline lower limb pain are shown in Figure 1A, whereas Figure 1B shows those with baseline lower limb pain. In general, all lower limb pain trajectories experienced an average increase in LMD over time; the only exception to this was the YYN trajectory, which experienced a slight decrease (0.07 logits). Those with lower limb pain at all 3 time points had a qualitatively different LMD trajectory to all other pain groups, with a much higher level of disability. At any given time point, those with current lower limb pain had higher levels of LMD at baseline than those with no lower limb pain. It was also true that those with lower limb pain at more time points overall had higher LMD scores at baseline than those with fewer time points with lower limb pain (Table 3). Changes in LMD from baseline to 3-year follow-up and 3- to 6-year follow-up were different in different lower limb pain trajectory groups. Those with lower limb pain at all 3 time points showed a consistently different pattern of LMD to all other trajectories: LMD levels were considerably higher and increased gradually over time. In general, there were larger increases in LMD with the onset of lower limb pain. This was seen when the onset was between baseline and 3-year follow-up and between 3- and 6-year follow-ups. Similarly, where there were recoveries from lower limb pain, there were relative reductions in LMD. For example, those in the trajectory NYN had a mean LMD score 0.43 logits higher than the NNN trajectory at baseline. With the onset of pain at 3-year follow-up, this increased to a 1.06-logit difference, but on recovery from pain at 6-year follow-up, this difference had returned to 0.34 logits.

When considering those free of lower limb pain at 3-year follow-up (Table 4), those experiencing an onset of lower limb pain at 6-year follow-up had, on average, LMD scores significantly higher at 6-year follow-up than those continuing to be free of pain in the lower limb. Adjustment for baseline sociodemographic factors and the concurrent onset of potentially confounding comorbid diseases minimally attenuated this association. When looking at the characteristics of the onset of lower limb pain, there was a dose-response relationship with increasing LMD and the onset of pain in multiple lower limb joints, more frequent pain, and more intense pain. Again, adjustment for baseline sociodemographic factors and the concurrent onset of selected comorbidities made only minor changes to these associations.

## 4 Discussion

### 4.1 Principal findings

Our findings suggest an independent, specific, potentially reversible, dose-response relationship between lower limb pain and LMD in community-dwelling adults age ⩾50 years. Against the background of a gradual increase in mean LMD levels over 6 years, the onset of lower limb pain accelerated this deterioration in function, whereas recovery from lower limb pain was associated with relative deceleration. The more widespread, frequent, or intense the lower limb pain, the more marked the decline in locomotor function. This pattern of associations remained after adjusting for several potential confounders.

### 4.2 Strengths and weaknesses

This study builds on previous work examining the association between lower limb pain and LMD (e.g., [19,25,27,42]) but goes further by measuring LMD on a continuum and examining changes in the presence of pain. Furthermore, the analysis of subsets of the data has allowed a more detailed investigation of characteristics of lower limb pain and changes in the level of LMD. However, the study also has some limitations that deserve consideration. Attrition from the sample over 6 years of the NorStOP was high, with only 25% of the original sample remaining at the end of the study. There was some evidence that those people who remained in the study were not representative of the group of baseline responders from which they were drawn: they were younger, more likely to be female, from higher socioeconomic groups, and in better health. Although this may bias the distribution of LMD scores and the prevalence of lower limb pain, the association between LMD and lower limb pain are likely to be less affected.

Associations between changes in pain status, LMD, and death were not investigated in this report. Although interesting and important, exclusion of known deaths should not bias our estimates of the association between pain and LMD. Mortality analyses were not undertaken because we do not know the complete vital status of nonresponders.

The analyses presented here considered the possible confounding effects of a number of morbidities on the association between the onset of lower limb pain and changes in LMD. These morbidities have previously been associated with LMD [27], and were associated with the presence of lower limb pain in the NorStOP sample. Adjustment was made only for the onset of morbidities, rather than their presence, because although they have previously been shown to have a cross-sectional association with LMD, this was not of interest here. By adjusting as we did for the onset of these conditions, we have ensured that it is not these morbidities that were responsible for the increase in LMD seen with the onset of pain. The self-reported nature of these morbidities could be a cause for concern. However, the number of self-reported conditions has wide provenance as a population marker of multiple morbidity and poor general health [12]. To ensure that our findings were robust, we conducted a complementary analysis in a subset of the NorStOP cohort who had consented to medical record review. Adjustment for the onset of chronic obstructive pulmonary disease and coronary heart disease, rather than the number of self-reported morbidities, did not provide different conclusions.

Although the new measure of LMD used in this study can be considered an improvement over previously used definitions of LMD, it still has limitations that deserve consideration. Because it is formed from only 5 items, taken from the SF-36 physical functioning subscale [43], it does not cover the whole range of functioning that might be seen in a population: it is clear that there are many people in the NorStOP cohort who have higher levels of functioning than can be measured by the scale and possibly also people who have less functioning than can be detected. This has, to a certain extent, been accounted for by the use of Tobit modelling in analyses, but is nevertheless a weakness of the measure. Furthermore, the items are not evenly spread across the range of disability that the scale covers. In combination, these issues suggest that the scale has a limited ability to sensitively measure changes in the underlying construct of LMD [30].

### 4.3 Comparison with the existing literature

The majority of previous studies have considered the association between prevalent musculoskeletal conditions and the subsequent onset of LMD over some fixed time period [10,11,15,19,25,42]. These studies have considered LMD to be a dichotomous phenomenon (disabled versus not disabled) (e.g., [15,19,42]). Although this definition may be necessary for decision-making purposes, it does not seem clinically plausible [34] and it has limited the full picture of the epidemiology of this common form of disability.

Shah et al. [33] have performed what is probably the most detailed analysis of the association between pain and LMD available in the literature. They showed a dose-response association between the number of joint pain sites at baseline and the onset of LMD (reduced gait speed) over a 14-year period.

Our study builds on these previous findings of an association between prevalent pain and the onset of LMD in two ways. First, we utilised a continuous measure of LMD, which permitted changes in the level of LMD over time to be detected across a wide spectrum of function in the community. Second, by examining 3 time points over the course of 6 years, this study is the first to assess fluctuations in lower limb pain status and their association with LMD.

### 4.4 Meaning

The association between the onset of lower limb pain and increases in LMD provide further support for the direct role of lower limb pain in the progression of LMD. Although recovery from pain resulted, on average, in a decrease in LMD, this group did not start from or return to the same level of LMD reported in the equivalent group of people who never experienced this pain. We may be observing the cumulative effect of recurrent episodes of pain on the underlying rate of decline in LMD. The failure of those recovering from pain to return to the LMD levels seen in those who never experienced the pain suggests a need for the primary prevention of lower limb pain. Although the prevention of all lower limb pain may be an unachievable target, the results of this study do provide evidence in favour of the treatment of lower limb pain, as well as rehabilitation of function in those presenting with disabling pain. Furthermore, the results are suggestive of a policy of identifying and targeting those with pain in order to prevent a cumulative effect of incomplete LMD recovery over time leading to a substantially larger amount of disability than in the pain-free population.

Our study does not provide information enabling us to examine in detail the many different mechanisms by which lower limb pain might increase the rate of deterioration in self-reported locomotor disability. These include conscious and unconscious avoidance and modification of activities in direct response to actual or anticipated pain and related symptoms on mobility, a pathway mediated by deficits in executive function attributed to pain, and reduced capacity relating to muscle weakness secondary to arthrogenic muscle inhibition and atrophy. In addition, conceptual overlap between pain on activities and the subjective rating of limitation and the effect of pain on negative appraisal of function may also contribute to the observed relationship.

### 4.5 Future work

Using long intervals between measurements will underestimate the true incidence of episodes of LMD and lower limb pain. Given the dynamic nature of both, future studies using shorter intervals (e.g., monthly measurements per Wolf and Gill [44]) would further our understanding of the interrelationship between episode characteristics of lower limb pain and their effect on LMD. It would also seem prudent to further assess the characteristics of those recovering from pain; in particular, those people recovering from pain and experiencing a recovery of function to levels similar to those in people without pain at any point. Also, the association between the characteristics of pain before a recovery and the level of change in LMD concurrent with that recovery deserves attention. However, the small numbers of individuals with this pattern of lower limb pain in the current study did not allow this to be investigated.

### 4.6 Conclusions

Changes in lower limb pain status are associated with changes in the level of LMD experienced by this sample of community-dwelling adults age ⩾50 years. This association is potentially causal: there are expected effects of the onset of and recovery from pain, a dose-response association, and little change in strength of association with adjustment for potentially confounding factors. There is evidence, seen in the partial recovery from lower limb pain relative to those remaining pain-free, that there is a cumulative effect of recurrent episodes of lower limb pain on LMD. Hence, primary or secondary prevention of lower limb pain and its underlying determinants (e.g., osteoarthritis) have the potential to reduce the number of years lived with disability in older populations.

## Conflict of interest statement

The authors have no conflicts of interest to declare.

## Figures and Tables

**Supplementary Figure 1:**
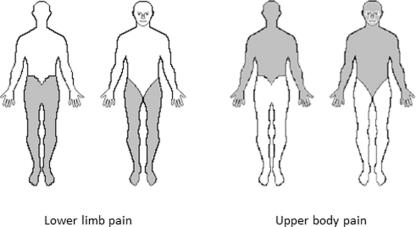


**Table 1:** Descriptive characteristics of the sample by lower limb pain trajectory.

Trajectory⁎	n 2506	Mean age (SD)	Female (%)	School education only† (%)	Manual occupation‡ (%)	Perceived inadequate income§ (%)
NNN	341	62.0 (8.6)	53.3	83.4	50.5	24.1
NNY	192	62.8 (8.1)	53.7	83.5	55.9	30.7
NYY	237	62.5 (8.3)	55.3	81.3	59.6	36.6
NYN	150	61.8 (8.1)	50.0	78.4	50.7	30.2
YNN	139	62.0 (9.3)	50.4	82.6	52.0	32.4
YYN	172	62.0 (8.2)	57.6	86.5	61.0	40.1
YNY	137	62.1 (8.4)	58.7	86.4	60.1	34.1
YYY	1138	63.1 (8.4)	59.4	88.1	62.4	48.1

⁎ N indicates no lower limb pain, Y indicates presence of lower limb pain. Ordering represents the 3 time points: baseline, 3-year follow-up, and 6-year follow-up. For example, NNY indicates no lower limb pain at baseline and 3-year follow-up and lower limb pain at 6-year follow-up; YYY indicates lower limb pain at all 3 time points.

† Alternative is having attended further education.

‡ Alternative is having a nonmanual occupation.

§ Alternative is perceiving income to be adequate.

**Table 2:** Response to the NorStOP Health Survey questionnaires over the 6 years of follow-up.

n (%)	Baseline sample	Not followed up	Sample for analysis
Overall⁎	5129	2623	2506
Gender			
Male	2354 (45.9)	1263 (48.2)	1091 (43.5)
Female	2775 (54.1)	1360 (51.9)	1415 (56.5)

Age group (y)			
50 to 59	1896 (37.0)	840 (32.0)	1056 (42.1)
60 to 69	1612 (31.4)	703 (26.8)	909 (36.3)
70 to 79	1173 (22.9)	721 (27.5)	452 (18.0)
⩾80	448 (8.7)	359 (13.7)	89 (3.6)

Educational attainment⁎			
Further education	619 (12.3)	258 (10.1)	361 (14.6)
School-age education only	4410 (87.7)	2304 (89.8)	2106 (85.4)

Occupational class⁎			
Nonmanual	1672 (37.2)	740 (32.9)	932 (41.5)
Manual	2827 (62.8)	1513 (67.2)	1314 (58.5)

Perceived adequacy of income⁎			
Adequate	2171 (43.0)	1201 (46.8)	1508 (60.9)
Inadequate	2876 (57.0)	1368 (53.3)	970 (39.1)

SF-12 score at baseline⁎^,^†			
Physical component summary	41.1 (12.6)	39.1 (12.7)	43.2 (12.1)
Mental component summary	49.1 (11.3)	48.2 (11.5)	50.0 (11.0)

Baseline sample: subjects provided LMD and lower limb pain data at baseline and consented to further contact. Not followed up: subjects were included in baseline sample, but did not respond at all 3 time points, or responded but did not provide LMD and lower limb pain data at all 3 time points. Sample for analysis: subjects provided LMD and lower limb pain data at all 3 time points. LMD = locomotor disability; NorStOP = North Staffordshire Osteoarthritis Project; SF-12 = Short Form 12.

⁎ Available only for those individuals responding at baseline and subject to missing data.

† Mean (SD).

**Table 3:** Locomotor disability scores at baseline and at 3- and 6-year follow-ups by lower limb pain trajectory: results from the Tobit regression modelling.

	Mean locomotor disability (logits) (95% confidence interval)		
Trajectory⁎	Baseline†	3-year follow-up‡	6-year follow-up§
Overall	1.28 (1.13–1.44)	1.80 (1.69–1.89)	2.17 (2.07–2.28)
NNN	−1.46 (−1.90–1.02)	−0.51 (−0.82–−0.21)	−0.18 (−0.47–0.11)
NNY	−0.67 (−1.22–−0.16)	0.04 (−0.33–0.42)	1.01 (0.65–1.36)
NYY	0.05 (−0.41–0.50)	1.27 (0.95–1.59)	1.80 (1.50–2.11)
NYN	−1.03 (−1.63–−0.42)	0.55 (0.14–0.96)	0.16 (−0.27–0.58)
YNN	0.22 (−0.38–0.83)	0.30 (−0.15–0.75)	0.76 (0.33–1.20)
YYN	1.33 (0.82–1.84)	1.45 (1.07–1.82)	1.24 (0.87–1.62)
YNY	1.06 (0.48–1.64)	0.67 (0.23–1.11)	1.42 (1.00–1.83)
YYY	3.33 (3.13–3.53)	3.54 (3.40–3.68)	3.98 (3.84–4.12)

⁎ N indicates no lower limb pain, Y indicates presence of lower limb pain. Ordering represents the 3 time points: baseline, 3-year follow-up, and 6-year follow-up. For example, NNY indicates no lower limb pain at baseline and 3-year follow-up and lower limb pain at 6-year follow-up; YYY indicates lower limb pain at all 3 time points.

† Adjustment is made for age group, gender, baseline socioeconomic status (educational attainment, occupational class, and perceived adequacy of income).

‡ Adjustment is made for age group, gender, socioeconomic status, and LMD at baseline, in fractional polynomial form (0.5, 1).

§ Adjustment is made for age group, gender, socioeconomic status, and LMD at 3-year follow-up, in fractional polynomial form (0.5, 1).

**Table 4:** LMD at 6-year follow-up in those without lower limb pain at 3-year follow-up: adjustment for the concurrent onset of comorbidities and lower limb pain characteristics at 6 years.

	β (95% CI)⁎	β (95% CI)†	β (95% CI)‡
Any lower limb pain	0.82 (0.44–1.20)	0.77 (0.38–1.16)	0.67 (0.26–1.08)
Number of pain sites (0 to 3)§	0.36 (0.14–0.57)	0.37 (0.14–0.59)	0.26 (0.03–0.48)

Chronicity of pain			
No days (n = 595)	0	0	0
1 to 30 days (n = 971)	0.48 (0.06–0.90)	0.42 (0.00–0.85)	0.39 (−0.03–0.82)
31 to 89 days (n = 98)	0.39 (−0.21–0.99)	0.66 (0.05–1.28)	0.50 (−0.12–1.11)
⩾90 days (n = 73)	1.44 (0.79–2.10)	1.61 (0.92–2.29)	1.44 (0.94–2.13)

Intensity of pain (0–10 NRS)§	0.19 (0.13–0.26)	0.20 (0.13–0.27)	0.19 (0.12–0.26)

β = regression coefficient from Tobit model; CI = confidence interval; LMD = locomotor dysfunction; NRS = numerical rating scale.

⁎ Values adjusted for LMD score at 3-year follow-up in fractional polynomial form (0.5, 1).

† Values adjusted for LMD score at 3-year follow-up in fractional polynomial form (0.5, 1), age group, gender, socioeconomic status (educational attainment, occupational class, and perceived adequacy of income).

‡ Values adjusted for LMD score at 3-year follow-up, in fractional polynomial form (0.5, 1), age group, gender, socioeconomic status, onset of chest problems, eyesight problems, leg pain on walking, depression, and pain in the upper body.

§ Modelled in linear form.
